# Violence among Adolescents in Qatar: Results from the Global School-based Student Health Survey, 2011

**DOI:** 10.7759/cureus.2913

**Published:** 2018-07-02

**Authors:** Mohamed O Bala, Mohamad A Chehab, Ayman Al-Dahshan, Salah Saadeh, Abdulhameed Al Khenji

**Affiliations:** 1 Community Medicine Residency Program, Hamad Medical Corporation, Doha, QAT; 2 Service Development Department, Primary Health Care Corporation, Doha, QAT

**Keywords:** qatar, global school-based student health survey, youth violence, adolescent health

## Abstract

Background

Despite being a neglected issue in adolescent health, interpersonal violence such as physical fighting constitutes a prominent cause of physical injuries in adolescents.

Aim

We aimed to study the prevalence of physical fighting and its associated factors among Qatar's adolescent population.

Method

We analyzed secondary data from Qatar's Global School-based Student Health Survey (GSHS) 2011 to determine the prevalence as well as the associated factors of being engaged in a physical fight in the last 12 months.

Results

It was found that almost half of the participants (49%) were involved in a physical fight; mostly males (60.5%) than females (37.6%). Being bullied, smoking, and having parental supervision were positively associated with physical fighting (OR = 3.97, 95% CI (3.68, 4.28); OR = 1.78, 95% CI (1.61, 1.97); OR = 1.14, 95% CI (1.05, 1.23), respectively).

Conclusion

Further behavioral research on adolescent violence will inform the development of youth-targeted violence prevention programs.

## Introduction

Physical injuries have become a global health issue, especially among adolescents. Fractures, open wounds, concussions, and burns represent leading causes of mortality, morbidity, and disability among the adolescent age group [1, 2]. One of the prominent causes of adolescent injury is interpersonal violence, which has increased significantly over the past years [3]. Globally, interpersonal violence represents the fifth cause of death in this age group and accounts for more than one-tenth (12%) of adolescent male deaths. In spite of that, it remains to be a neglected issue of adolescent health globally [4].

Violence is a costly public health issue and several studies, as well as governmental reports, have revealed that nations expend a substantial sum of resources in this regards. In the United States of America, the economic burden of interpersonal violence in 2013 has been estimated at 671 billion US$ as a result of lifetime medical and work loss costs. Among those aged 10-19 years, the total cost of fatal and nonfatal injuries amounted to approximately 64 billion US$ [5]. On the other hand, evidence regarding this matter in the developing world is scarce, where many of the available figures underestimate the actual burden of interpersonal violence. In addition, such risky behavior translates into social, emotional, and economic consequences for adolescents and their caregivers or families [6]. In comparison to global figures, the Eastern Mediterranean region has significant morbidity and mortality rates attributable to violence among the adolescent age group [7].

The Gulf Cooperation Council’s (GCC) six member-states are home to one of the most youthful communities in the world, with one-third to one-half of the population below 25 years of age. Additionally, individuals in this age group account for a large proportion of the population in Oman (51.5%), Saudi Arabia (50.8%), Bahrain (43.9%), Kuwait (37.7%), Qatar (33.8%), and the United Arab Emirates (31%) [8]. However, data is limited on the prevalence of interpersonal violence among adolescents in the GCC region as well as its associated factors. The absence of baseline data on the prevalence of violence as well as associated exposures hinders public health practitioners and policymakers from developing evidence-based violence prevention programs. Furthermore, the lack of knowledge on this issue in the region renders the advocacy for resource allocation to such programs a tiresome task [9].

Therefore, we carried out this study using data from Qatar’s 2011 Global School-based Student Health Survey (GSHS) to contribute to much-needed literature about adolescent violence in the region. This paper describes the prevalence and associated factors of physical fighting among Qatar’s adolescent population in grades 7th–9th.

## Materials and methods

This study was a secondary data analysis of GSHS, conducted in Qatar during 2011. The GSHS was developed by the World Health Organization (WHO) in collaboration with United Nations' UNICEF, UNESCO, and UNAIDS, and with technical assistance from the US Centers for Disease Control and Prevention, to generate data on health and social behavior among school-going adolescents. Qatar is located in Western Asia, occupying the western coast of the Arabian Gulf. According to the World Bank, Qatar is a high-income country with a population estimate of 2.57 million in 2016 [10].

GSHS sampling technique

The GSHS employs a two-phase probability sampling process. During the initial stage, schools represent the primary sampling units and are selected based on a probability proportional to their enrolment size. In the second phase, the investigators obtain a systematic sample of the classes at the selected school. All students present in the selected classes will be eligible to participate by completing a self-administered questionnaire on a voluntary basis.

Study hypothesis and variables

The study has three hypotheses; the first was that adolescents who had adequate parental supervision were less likely to be engaged in fights, the second was that females were less likely to be involved in fights, and the last was that smoking was associated with fighting. The list of independent variables included: age, gender, past history of bullying, past history of cigarette smoking, and past history of parental supervision. The dependent variable was a history of engagement in fights.

GSHS tool

The GSHS used a self-administered questionnaire that addresses the leading causes of mortality and morbidity among adolescents. Our study focused on the questions that measured the variables of interest; Q1 (how old are you?), Q2 (what is your sex?), Q26 (during the past 12 months, how many times were you in a physical fight?), Q31 (during the past 30 days, on how many days were you bullied?), Q45 (during the past 30 days, on how many days did you smoke cigarettes?). For the latter three questions, possible response options were provided with a range of 8-9 options.

Data analysis

The data was analyzed through the complex samples functions in SPSS software for Windows, version 16 (SPSS, Chicago, Illinois) according to the methods section of the GSHS Data User’s Guide. The level of significance was determined at <0.05. The absolute counts shown in this paper are unweighted. However, all the percentages and statistical tests are weighted according to three variables (primary sampling unit, stratum, and weight). Odds ratios (OR) and their associated 95% confidence intervals (CI) are employed to compare injury rates by age, sex, and other characteristics. Logistic regression analysis was conducted to estimate the association between relevant co-variates and physical fights. After which, the results of the studied factors were reported as adjusted odds ratios with 95% confidence intervals.

The data analysis compensated for different patterns of non-response. This was achieved through utilizing a weighting factor, which was calculated through the following formula:

W = W1 × W2 × f1 × f2 × f3 × f4

W1: the inverse of the probability of selecting a school

W2: the inverse of the probability of selecting the classroom

fl: non-response adjustment factor calculated by school size category (small, medium, large)

f2: class-level non-response adjustment factor calculated for each school

f3: student-level non-response adjustment factor calculated by class

f4: post-stratification adjustment factor calculated by grade

## Results

The number of Qatari adolescents who participated in Qatar’s 2011 GSHS reached 2,021, more than half of which were 13-years-old (1111, 53.8%), females (1075, 51.8%), and self-reported no history of being bullied (57.6%) in the last 30 days. The socio-demographic characteristics of those participants are presented in Table 1. Furthermore, most of the surveyed adolescents were non-smokers (79.8%) and under parental supervision (70.4%) during the last 30 days. In addition, almost half of the participants (49%) self-reported being involved in a physical fight during the past 12 months, mainly 60.5% of total males and 37.6% of total females.

**Table 1:** Selected characteristics of male and female adolescents in the Global School-based Student Health Survey in Qatar, 2011 - % (Weighted), N (Un-weighted).

Characteristics	Total % (N = 2021)	Males % (N = 912)	Females % (N = 1075)
Age (years):			
≤13	53.8 (1111)	44.6 (420)	61.7 (669)
14	28.8 (564)	29.0 (259)	28.9 (301)
15	13.6 (256)	21.1 (185)	6.8 (69)
≥16	3.9 (69)	5.2 (40)	2.6 (27)
Gender:			
Female	51.8 (1075)	-	-
Male	48.2 (912)	-	-
Bullied in the last 30 days:			
No	57.6 (1067)	50.7 (417)	64.8 (642)
Yes	42.4 (771)	49.3 (402)	35.2 (348)
Smoking cigarettes in the last 30 days:			
No	79.8 (1482)	72.0 (578)	86.9 (882)
Yes	20.2 (364)	28.0 (226)	13.1 (130)
Parental supervision in the last 30 days:			
No	29.6 (519)	32.5 (250)	27.0 (260)
Yes	70.4 (1252)	67.5 (516)	73.0 (717)
In fight in the last 12 months:			
No	51.0 (1010)	39.5 (340)	62.4 (661)
Yes	49.0 (938)	60.5 (532)	37.6 (384)

The OR, with a 95% CI, of the fitted logistic regression, are shown in Table 2. It was found that adolescents aged 14 years were more likely to engage in a fight over the last 12 months, when compared to their peers of other age groups (OR = 1.37, 95% CI (1.26, 1.49)). In addition, male participants were 1.4 times more likely to be involved in a fight than their female counterparts (OR = 2.4, 95% CI (2.23, 2.60)). On the other hand, being bullied (victimization) had the strongest positive association with physical fight overall (OR = 3.97, 95% CI (3.68, 4.28)) as well as among males (OR = 3.51, 95% CI (3.14, 3.94)) and females (OR = 4.22, 95% CI (3.80, 4.68)). Also, it was found that adolescents who reported smoking cigarettes within the past month were more likely to be engaged in fights (OR = 1.78, 95% CI (1.61, 1.97); more so among females (OR = 2.5, 95% CI (2.12, 2.93)) than their male peers (OR = 1.38, 95% CI (1.22, 1.57)). Finally, parental supervision was slightly associated with physical fighting (OR = 1.14, 95% CI (1.05, 1.23)), especially among the male respondents (OR = 1.60, 95% CI (1.43, 1.80)).

**Table 2:** Relationship between physical fighting and selected factors among male and female adolescents in Qatar, 2011.

	Odds ratio (OR) with 95% confidence interval (CI)		
	Total	Males	Females
Age (years):			
≤13	1.00	1.00	1.00
14	1.37 (1.26, 1.49)	1.23 (1.09, 1.40)	1.45 (1.30, 1.62)
15	1.22 (1.08, 1.37)	1.16 (1.01, 1.34)	1.16 (0.95, 1.43)
≥16	0.50 (0.41, 0.61)	0.27 (0.21, 0.35)	1.31 (0.98, 1.74)
Gender:			
Female	1.00	-	-
Male	2.40 (2.23, 2.60)	-	-
Bullied in the last 30 days:			
No	1.00	1.00	1.00
Yes	3.97 (3.68, 4.28)	3.51 (3.14, 3.94)	4.22 (3.80, 4.68)
Smoking cigarettes in the last 30 days:			
No	1.00	1.00	1.00
Yes	1.78 (1.61, 1.97)	1.38 (1.22, 1.57)	2.50 (2.12, 2.93)
Parental supervision in the last 30 days:			
No	1.00	1.00	1.00
Yes	1.14 (1.05, 1.23)	1.60 (1.43, 1.80)	0.84 (0.75, 0.94)

## Discussion

The current paper describes the prevalence of physical fighting and its associated factors among school-going adolescents in the State of Qatar. It was shown that almost half (49%) of the participating students were involved in physical fighting during the past year; more so among boys (60.5%) than girls (37.6%). This prevalence is comparable to the Eastern Mediterranean regional average of 46.73% but much higher than that reported in the Youth Behavioral Risk Survey (31.41%, 2009) of the United States [11]. Despite Qatar being one of the highest-income countries in the world [12], the relatively high prevalence of fighting among its adolescents comes in contrast with previous research that established a significant association between low socio-economic status and violence [13-15]. Moreover, a gender-bias towards males regarding issues of interpersonal violence and physical fighting has been reported extensively in earlier studies as well as international reports [11, 16]. Thus, when put in social context and cultural norms, Qatari male adolescents appear geared towards reflecting masculinity and toughness among their peers and family members.

In the current study, less than half (42.4%) of the respondents reported being victims of bullying during the past month. This result is relatively high when compared to that of low- and middle-income nations as well as high-income countries [17, 18]. In addition, bullying in the current study was more common among males (49.3%) than females (35.2%), which is similar to reports from other countries as Ethiopia, India, and Vietnam. Moreover, males were subject to different types of bullying than females, where the former were subjected to verbal and physical bullying while the latter were subject to indirect bullying [19]. Similarly, a study by Wang et al. examined the prevalence and associated factors of four types of bullying among US adolescents and found that physical and verbal bullying was more prevalent among boys, countered by relational bullying among girls. Additionally, the researchers reported on cyberbullying, where boys were mainly designated as cyber bullies, while girls were mostly the cyber victims [18]. However, only verbal and physical bullying data is collected through the GSHS questionnaire, while other forms of bullying are designated as “I was bullied in some other way”. Thus, there is a need for further expanding of the GSHS multiple-choice answers for questions on bullying because cyberbullying has emerged as a global health issue after the rise of social media platforms and associated high-tech behavior among adolescents [20].

Factors that were associated with physical fighting among students during the last 12 months included being bullied and smoking. These results are in concordance with those reported by Muula et al. on bullying and fighting among Venezuelan adolescents, where a significant dose-response relationship coupled bullying with physical fighting [21]. Likewise, a study by Rudatsikira et al. among Chilean adolescents revealed that substance use (cigarette smoking, drinking alcohol, drugs) and being bullied were positively associated with physical fighting [22]. Consequently, bullying and drug abuse interact in a larger vicious cycle and may lead to cognitive and non-cognitive harm such as depression, stress, lack of satisfaction with life, and academic impairment [23].

The current study identified a slight but statistically significant positive association between parental supervision and physical fighting among school students, which contradicts most of the available literature on this issue that identified parental supervision as a protective factor against physical fighting [9, 18, 21, 22]. Furthermore, this finding is more pronounced among male respondents (OR = 1.60, 95% CI (1.43, 1.80)). Adolescents identify their parents as significant individuals in their lives, with boys siding with their fathers and girls with their mothers [24]. Thus, the style of parenting affects adolescents’ behavior inside and outside their home and propagates onto adulthood, with physical punishment increasing the risk of fighting, bullying, and victimization among sons and daughters [25]. Furthermore, much research has focused on the importance of parenting style in yielding positive outcomes among adolescents, with a variation of one style’s effect because of cultural and contextual factors. One study suggested designing family-centered prevention programs targeting parents and adolescents as well as public health policies that mitigate any contextual factors [26]. However, research has also shown that peer pressure facilitates violence among adolescents, who are more prone to become violent when such behavior has been demonstrated by a friend or a friend of a friend [27].

The GSHS is a self-reporting questionnaire and has several limitations as recall bias. Another limitation is that certain exposures in the survey might have been misreported due to cultural barriers, social norms, fear of stigma, and social desirability bias. Additionally, the GSHS is cross-sectional, making temporality and causality difficult to infer. Furthermore, the results of such survey might not be generalizable onto the adolescent population of Qatar because it involves only school-going youth, despite the fact that the majority of the country’s adolescent population are enrolled in schools.

## Conclusions

The current study aimed at determining factors associated with physical violence among school-going adolescents in the State of Qatar. Several associated factors were identified such as smoking, bullying, and parental supervision. This issue is of national and regional public health importance and demands a multisectoral and multidisciplinary collaboration to address the social and environmental factors behind it. In addition, there is a need for further behavioral researches to fill the knowledge gaps which will build robust evidences for planning of youth-targeted violence prevention programs in relevant settings such as schools and other community settings.
